# Urine mercury levels correlate with DNA methylation of imprinting gene *H19* in the sperm of reproductive-aged men

**DOI:** 10.1371/journal.pone.0196314

**Published:** 2018-04-26

**Authors:** Zhaoxu Lu, Yufeng Ma, Linying Gao, Yingjun Li, Qiang Li, Mei Qiang

**Affiliations:** 1 Department of Child and Adolescence Health, School of Public Health, Shanxi Medical University, Shanxi, Taiyuan, China; 2 Department of Sanitary Inspection, School of Public Health, Shanxi Medical University, Shanxi, Taiyuan, China; 3 Department of Andrology, Children’s Hospital and Women Health Center of Shanxi, Shanxi, Taiyuan, China; University of Bristol, UNITED KINGDOM

## Abstract

**Background:**

Mercury (Hg) is a well-recognized environmental pollutant known by its toxicity of development and neurotoxicity, which results in adverse health outcomes. However, the mechanisms underlying the teratogenic effects of Hg are not well understood. Imprinting genes are emerging regulators for fetal development subjecting to environmental pollutants impacts. In this study, we examined the association between preconceptional Hg exposure and the alteration of DNA methylation of imprinting genes *H19*, *Meg3*, and *Peg3* in human sperm DNA.

**Methods:**

A total of 616 men, aged from 22 to 59, were recruited from Reproductive Medicine Clinic of Maternal and Child Care Service Center and the Urologic Surgery Clinic of Shanxi Academy of Medical Sciences during April 2015 and March 2016. Demographic information was collected through questionnaires. Urine was collected and urinary Hg concentrations were measured using a fully-automatic double-channel hydride generation atomic fluorescence spectrometer. Methylation of imprinting genes *H19*, *Meg3* and *Peg3* of sperm DNA from 242 participants were examined by bisulfite pyrosequencing. Spearman’s rank and multivariate regression analysis were used for correlation analysis between sperm DNA methylation status of imprinting genes and urinary Hg levels.

**Results:**

The median concentration of Hg for 616 participants was 9.14μg/l (IQR: 5.56–12.52 μg/l; ranging 0.16–71.35μg/l). A total of 42.7% of the participants are beyond normal level for non-occupational exposure according to the criterion of Hg poisoning (≥10 μg/L). Spearman’s rank analysis indicated a negative correlation between urinary Hg concentrations and average DNA methylation levels of imprinted genes *H19* (r_s_ = −0.346, p <0.05), but there was no such a correlation for *Peg3* and *Meg3*. Further, we analyzed the correlation between methylation level at individual CpG site of *H19* and urinary Hg level. The results showed a negative correlation between urinary Hg concentrations and three out of seven CpG sites on *H19* DMR, namely CpG2 (r_s_ = −0.137, p <0.05), CpG4 (r_s_ = −0.380, p <0.05) and CpG6 (r_s_ = −0.228, p <0.05). After adjusting age, smoking, drinking, intake of aquatic products and education by multivariate regression analysis, the results have confirmed the correlation as mentioned above.

**Conclusions:**

Mercury non-occupational environmental exposure in reproductive-aged men was associated with altered DNA methylation outcomes at imprinting gene *H19* in sperm, implicating the susceptibility of the developing sperm for environmental insults.

## Introduction

Mercury (Hg) is a common environmental contaminant that derives from anthropogenic sources like coal-burning emissions, industrial waste, and accumulates through ocean and soil. The toxic effects of Hg on human have been well documented for the past decades regarding the aspects of metabolism, behavioral, neurochemical, hormonal, and reproductive changes [1–3]. The effect of mercury toxicology differs with respect to different forms of mercury, to which people exposed. The target organ for methylmercury (MeHg), which most probably due to frequent and large amount fish consumption is the brain; target organs for elemental mercury usually in vapor are the brain and kidney; for inorganic Hg in the form of compounds targets the kidney[4]. In addition, both MeHg and elemental mercury (not inorganic Hg), can also pass the placental barriers[4], therefore impact fetal growth. Recently, a case-control study of neural tube defects (NTDs) found that placental concentrations of Hg were significantly higher in the mothers who had NTDs children compared to those who had normal children. This study for the first time revealed an 8.8-fold increase in risk of NTDs in associated with placental high Hg concentrations [5]. In particular, in this study, data comes from Shanxi, an inland area of China, where fish is not main meat consumed by local residents, while coal-processing waste products spreading in the air and soil are the main pollution sources. The exposure of Hg settled in the environment is low, however dredging up sediments can increase exposure elemental or inorganic mercury. Previous study indicated that urine Hg reflects primarily the inorganic or elemental exposure [4]. Although, major route of mercury excretion is urine and faeces, study for eliminating mercury showed major in the urine and however, the amount in the feces is rarely [6].

Intergenerational impacts of prenatal exposure to adverse environmental pollutants have recently received much attention. Moreover, exposures to environmental toxic metals prior to pregnancy may alter fetal development and lead to adverse health outcomes [7]. In the body, Hg not only crosses placental barrier to directly damage the early embryonic development, but also readily crosses the blood-testis barrier to inhibit spermatogenesis and decrease sperm capacitation [5,8]. Interestingly, an animal experiment found that methylmecury is easy to accumulate in the testicles, induce testicular pathological changes and decreasing mating rate of male rats, female pregnancy rate, the average number of live births and lower development levels of fetus in methylmecury exposure group than in blank control group [9]. In sum, these studies suggest a role of paternal environmental exposure on fetal development. Although studies have revealed the association of paternal exposure to Hg with adverse pregnant outcome, but the mechanism of paternal exposure to Hg inducing abnormal pregnancy outcome is not well understood.

DNA methylation is an important epigenetic mechanism being involved in early embryonic development in mammalian. A variety of environmental factors such as toxicants, nutrients and drug of abuse has shown to induce alterations in the epigenetic markers which were associated to multi-generational inheritance effects [10]. In the past decade, much attention has been focused on the impact of prenatal maternal environmental exposure on imprinting genes and on next generation. However, the epigenome is considered to be very susceptible to environmental toxicants during spermiogenesis as well. Benchaib et al. analyzed global DNA methylation status in sperm samples of 63 cases found that global DNA hypomethylation is closely related to abnormal pregnancy outcome [11]. In addition, a study in rat provided evidence of a link between abnormal DNA methylation in sperm and altered fertility, abnormal embryo development [12]. Further, animal studies reveal environmental exposure to toxic metals, including Hg, can decrease sperm DNA methylation and increase in infertility through generation of oxidative stress on testicular [8,13]. In particular, imprinted genes are monoallelically expressed in a parent-of-origin dependent manner and controlled by parental allele-specific differentially methylated regions (DMRs) established in gametes during early development and this methylation pattern is largely maintained in somatic tissues. Animal experiments found that mercury is easy to accumulate in the testicles, which resulted in inhibit DNA methyltransferase activity in spermatozoa through Hg induced oxidative stress, such as demethylation of the regulatory sequences of H19 and Gtl2/Dlk1 in male germ cells [8,14,15]. Although DNA methylation alterations at these regulatory regions has been associated with the early embryonic development [16], human data linking paternal exposure to Hg and the alterations of DNA methylation of imprinting genes in sperm are sparse. For this regard, in the present study, we examined the correlation between paternal urinary Hg levels and the methylation level on the DMRs of three imprinted genes, namely, *H19*, *Peg3* and *Meg3* in human sperm, which previously have been found to be associated with fetus development [17].

## Materials and methods

### Study participants and data collection

In this study, 616 reproductive-age males were recruited from reproductive medical clinic of Children’s Hospital and Women Health center in Shanxi and Shanxi Academy of Medical Sciences in April 2015 and March 2016. The participants visited for the purpose of either pre-pregnant check or family infertility examination. Upon enrollment, they were asked to complete a questionnaire including social-demographic factors (age, occupation, education, family history of birth defects); lifestyle, and dietary preference. Those who have family history of birth defects were excluded. Written informed consent was obtained from the participants. In addition, all study protocols were approved by the Shanxi Medical University Institutional Review Board.

### Urine and semen collection

Fifty milliliter midstream urine was collected on site with clean plastic containers. At the same day, semen samples were obtained by requiring participants abstain from ejaculation for at least 3 days, but no more than 7 days prior to their visit. Semen was collected on site by masturbation without the use of lubricants into a sterile polypropylene collection container. After liquefaction, semen samples were centrifuged at 200×g for 15 min to pellet sperm. Both urine and sperm were stored at -80 °C for further analyses.

### Measurement of urine Hg concentrations

To determine the levels of internal exposure of environmental, urine total Hg concentrations were evaluated in 618 males. The assay was carried out as previously described [18] with minus modification (http://dx.doi.org/10.17504/protocols.io.n5tdg6n). First, the urine samples were digested using the bromination procedure [19]. Briefly, 2 ml of urine was used 8 mol l^-1^ nitric acid, anti-foaming agent (a few drops) and 0.0599mol l^-1^ potassium bromate-0.0234mol l^-1^ potassium bromide reagent, were added respectively. The tubes were tightly capped and allowed to stand on bench for 20 min to complete breakdown of organic Hg compounds. Afterward, 10ml doubly deionized water (DDW) was added and then a few drops of 1.439mol l^-1^ hydroxylamine hydrochloride was added to remove free bromine after digestion. Finally, DDW was added to fill the volume up to 25 ml. Notably, frozen (-80°C) urine was equilibrated to room temperature for 2 hours, then homogenized with a slow shaker for 30 minutes and all reagents used were of guaranteed reagent and all solutions were prepared in DDW with a minimum resistivity of 18.0 MΩ cm obtained from a Heal Force water purification system (Heal Force, Biomedic Inc., Hong Kong). Besides, the solutions were prepared daily.

Following digestion, Hg fluorescent intensity was assayed by using a fully-automatic double-channel hydride generation atomic fluorescence spectrometer (AFS-9700, Beijing Haiguang Instrumentals Co. Ltd., Beijing, China). The Hg concentrations were obtained utilizing standard-curve method. To avoid possible contamination during the digestion procedure and sample manipulation, a blank solution was carried through the preparation and device assay process along every twenty samples. All calibration regression curves had correlation coefficients as >0.999. Certified standards of Hg (GSB 04-1729-2004) were purchased from National Reference Materials Co. (Beijing, China). Spiked recovery test was performed to ensure the precision and accuracy of the analyses. A sample of standard materials with known reference concentrations of Hg was prepared and carried through the preparation and assay processes along every twenty urine samples to verify the stability of the process. The limit of detection for Hg was 0.09μg/l. If a concentration of Hg was lower than the limit of detection, the concentration of the sample was recorded as zero.

### DNA extraction and bisulfite treatment

Genomic DNA extraction was performed combining a sperm lysis protocol of Modified Guanidinium Thiocyanate Method [20] and QIAamp DNA micro kit (Qiagen, CA, USA) for isolating DNA. DNA was quantified using a *Nanodrop 2000* Spectrophotometer (Thermo Scientific).

Genomic DNA (1μg) was then treated with sodium bisulfite to convert unmethylated cytosine residues to uracil and leave methylated cytosine unchanged, using EZ Methylation Gold-Kit (Zymo Research, Orange, CA, USA) per the manufacturer’s instructions. The treated DNA was eluted in 15μl of TE buffer. Two microliters of the post bisulfite-treated DNA were used for subsequent PCR amplification.

### PCR amplification of bisulfite-treated sperm DNAs and subsequent pyrosequencing

Bisulfite converted DNA (~50 ng) was amplified by PCR using PyroMark PCR Kit (Qiagen, CA, USA). All reactions were performed using provided PCR mixtures (total volume at 25μl) with 0.2uM each of the forward and reverse PCR primers. Each reaction also contained 2.5 ul of CoralLoad Concentrate (10x) for checking amplicons on an agarose gel. Reverse primer was conjugated to biotin. These single stranded amplicons were isolated using the Pyrosequencing Work Station and underwent pyrosequencing on a Pyromark Q96 MD pyrosequencing instrument (Qiagen, CA, USA). PCR and pyrosequencing primers are provided in Table 1. The PCR conditions were used as following: 94 °C for 15 min, followed by 45 cycles of 94 °C, 30s, 56 °C, 30s, 72°C, 30s, and by a 72 °C final extension step for 10min.

**Table 1:** Primer sequences for PCR amplification.

DMR (Chr)	Forward primer	Reverse primer*	Sequencing primer
*H19* (11p15.5)	GTATATGGGTATTTTTTGGAGGT	ATATCCTATTCCCAAATAA	TGGTTGTAGTTGTGGAAT
*MEG3* (14q32.3)	GGGATTTTTGTTTTTTTTTGTAGTAGG	CCAACCAAAACCCACCTATAAC	TTTGGGGTTGGGGTT
*PEG3* (19q13.43)	TAATGAAAGTGTTTGAGATTTGTTG	CCTATAAACAACCCCACACCTATAC	GGGGGTAGTTGAGGTT

*Biotin tagged primer.

The biotinylated PCR products were purified using streptavidin-sepharose beads (Amersham, New Jersey, USA) and sequenced using PyroMark Gold Q96 kit (Biotage AB, Uppsala, Sweden). The degree of methylation at each CpG site was determined using PyroMark CpG Software (Biotage AB, Uppsala, Sweden). Pyrosequencing assays (http://dx.doi.org/10.17504/protocols.io.n52dg8e) were performed in duplicate in sequential runs (technical replicates), and the values shown represent the mean methylation for each individual CpG site. The number of CpGs sites analyzed at each DMR is as follows: *H19* (7 CpGs), *Meg3* (8 CpGs) and *Peg3* (7 CpGs) (for locations see the schematic diagram of the regions in S1 Fig).

### Statistical analysis

Medians and interquartile ranges (IQR) were employed to describe distributions of urinary Hg concentrations. Mann-Whitney U or Kruskal–ruskal test were used to compare the median levels of Hg between two groups or multiple social-demographic groups respectively. Spearman’s rank correlation analysis was used to determine the correlation between methylation status of 3 imprinting genes and urinary Hg concentrations, and multivariate regression models for further confirming the relationship between internal urinary Hg and methylation levels of the imprinting genes in sperm DNA under controlling potential confounders. A level of significance for all analyses was set at p < 0.05.

## Results

### Characteristics of study participants

A total 618 men aged ranging from 22 to 59 years were recruited in this study. Two of them, due to the value of urinary Hg was lower than the limit of detection (0.09μg/l), were excluded. The characteristics of 616 participants are presented in Table 2. They were all Han Chinese with an average age of 31. Of total, 53.7% have a college education or higher; 54.7% smoking; 30.0% drinking (details see questionnaire in S1 and S2 Tables); and 53.6% had higher self-reported intake aquatic products (fish and seafood).

**Table 2:** Social-demographic characteristics and urinary Hg levels.

	N(%)	Urinary Hg (μg/L)*					p-values
		Median	P_25_ ~ P_75_	Min	Max	Over-criteria ** n(%)	
Age							> 0.05
22–24	14(2.3)	8.84	6.48~13.86	1.17	16.28	6(42.9)	
25–29	224(36.4)	8.52	4.79~12.17	0.16	43.38	92(41.1)	
30–35	279(45.3)	9.36	5.88~13.31	0.68	71.35	123(44.1)	
36+	99(16.1)	8.76	5.10~11.73	0.45	31.42	42(42.4)	
Smoking							
Yes	337(54.7)	9.28	5.52~12.70	0.45	71.35	151(44.8)	> 0.05
No	279 (45.3)	8.94	5.65~12.43	0.16	59.40	112(40.1)	
Drinking							
Yes	185(30.0)	9.51	5.81~12.95	0.45	71.35	82(44.3)	> 0.05
No	431(70.0)	8.94	5.10~12.48	0.16	59.40	181(42.0)	
Aquatic products intake							
Yes	330(53.6)	8.74	4.84~12.19	0.16	59.22	130(39.4)	> 0.05
No	286(46.4)	9.47	6.31~13.09	0.45	71.35	133(46.5)	
Education							
Primary school	11(1.8)	8.44	5.35~11.73	3.21	31.42	3(27.3)	> 0.05
Middle school	147(23.9)	9.48	5.18~13.17	0.38	43.38	67(45.6)	
High school graduate	127(20.6)	7.80	4.62~11.51	0.45	71.35	46(36.2)	
College or above	331(53.7)	9.35	5.83~12.81	0.16	59.40	147(44.4)	
Total	616(100)	9.14	5.56~12.52	0.16	71.35	263(42.7)	

* Normalized by common specific gravity.

** Criterion of Hg poisoning: urinary Hg concentration ≥10 μg/L. US CDC. Specific Hazards/Chemical mergencies/ Mercury. Available from URL: https://emergency.cdc.gov/agent/mercury/mercelementalcasedef.asp

The median concentration of Hg for participants overall was 9.14μg/l (IQR: 5.56–12.52 μg/L; range = 0.16–71.35 μg/L). A total of 42.7% of these participants are beyond normal level for non-occupational exposure according to the criterion of Hg poisoning (urinary Hg concentration ≥10 μg/L, see Table 2). The results from Mann-Whitney U or Kruskal–ruskal test indicate no significant difference in median concentrations of Hg compared among different demographic groups (p > 0.05).

### Methylation status of imprinted genes in sperm DNA

**Fig 1:**
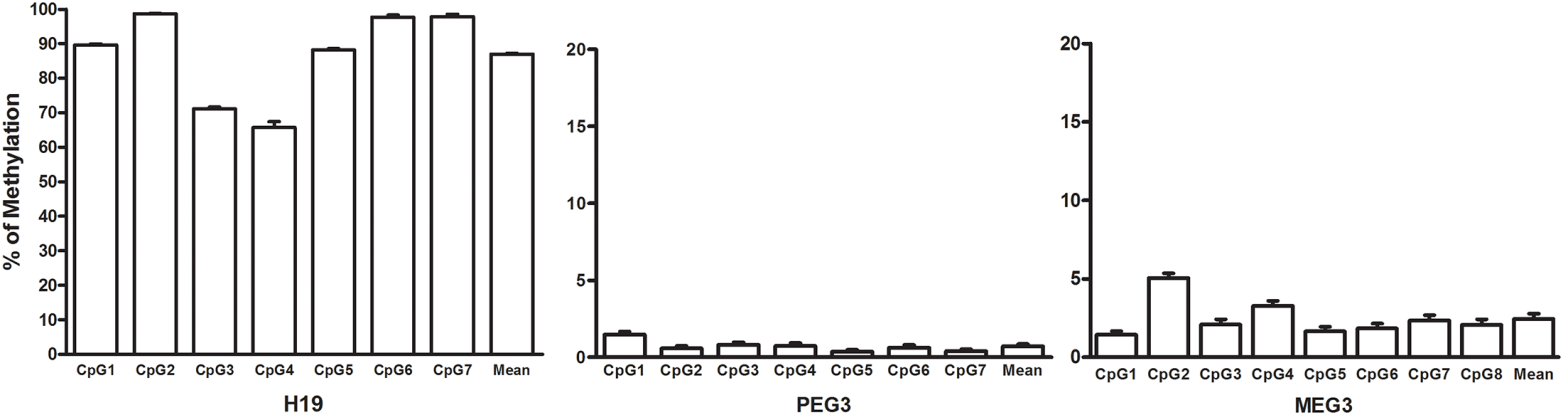
Levels of sperm DNA methylation at the DMRs of imprinted genes. The Bars represent percent of methylated cells by individual CpG site for each DMR of imprinting gene: *H19*, *Peg3* and *Meg3* as indicated. The results are the means ± SEM of 242 human sperm samples.

Two hundred and forty three out of 616 participants, who had sperm samples available, were included in the examination of methylation outcomes of the sperm DNA. The relationships presented in Table 2 were held amongst the 242 subjects (S3 Table). The methylation levels are expressed in percentage (Fig 1). As general expected, the mean methylation level of *H19* was 87.0%, being consistent with the known methylation range for the sperm DNA, i.e., closed to 100%; and the DMR for *Peg3* was almost unmethylated (0.7% in average) in these human sperm samples. One exception was the DMR for *Meg3*, which was unmethylated (close to 0%) in sperm, which is similar with a description of recent study in human sperm [21], despite being a known paternally methylated DMR (close to 100%) in mice [22].

### Correlational analysis between urinary Hg concentrations and DNA methylation

To examine the potential relationship between urinary Hg concentrations and DNA methylation levels of selected imprinted genes, Spearman’s rank correlation analysis was carried out by using average methylation level of selected CpGs in each gene. The results indicated that the average methylation levels of *H19* was negatively correlated with urinary Hg concentrations (r_s_ = −0.346, p <0.05) (Table 3). However, there was no such a correlation found in genes of Peg3 and Meg3. To further determine the specific impact of urinary Hg on individual CpG site on *H19* DMR, Spearman’s rank correlation analysis was then applied with methylation level at the individual site of each CpG. We therefore found that 3 out of 7 CpG sites on *H19* DMR, namely CpG2 (r_s_ = −0.137, p <0.05), CpG4 (r_s_ = −0.380, p <0.05) and CpG6 (r_s_ = −0.228,p <0.05), demonstrated a significant negative correlation between methylation levels and the levels of urinary Hg (Table 4).

**Table 3:** Spearman correlation analysis for average methylation levels of imprinted genes.

*H19*		*Peg3*		*Meg3*	
r_s_	p	r_s_	p	r_s_	p
−0.346	< 0.05	−0.011	> 0.05	+0.001	> 0.05

**Table 4:** Spearman correlation analysis for individual CpGs of *H19* methylation levels and urinary Hg.

	CpG1	CpG2	CpG3	CpG4	CpG5	CpG6	CpG7
r_s_	−0.035	−0.137	−0.035	−0.380	−0.066	−0.228	−0.080
p	> 0.05	< 0.05	> 0.05	< 0.05	> 0.05	< 0.05	> 0.05

Due to many environmental factors could contribute the dynamic alteration of DNA methylation, we therefore selected co-variables including age, smoking, drinking, intake of aquatic food and education based on known or observed associations with DNA methylation at these or other aspects with paternal risk factors. Multivariate procedures were used by above described co-variables to control potential confounding and accounting for cluster effects at the individual CpGs. By multivariate regression analysis, the results indicate a similar correlation, i.e., a significant negatively correlated between urinary Hg concentrations and the methylation levels of *H19* at both average level (β = −0.321, se = 0.027; p <0.05) and three individual CpGs, i.e., CpG2 (β = −0.129, se = 0.048; p <0.05), CpG4 (β = −0.414, se = 0.166; p <0.05) and CpG6 (β = −0.219, se = 0.076; p <0.05) of H*19* DMRs (Table 5).

**Table 5:** Multivariate regression analyses of imprinted genes *H19*, *Peg3* and *Meg3* and urinary Hg.

CpG Sites	*H19*			*Peg3*			*Meg3*		
	β	SE	p	β	SE	p	β	SE	p
CpG1	−0.063	0.054	> 0.05	−0.011	0.020	> 0.05	+0.021	0.027	> 0.05
CpG2	−0.129	0.048	< 0.05	−0.007	0.016	> 0.05	+0.033	0.033	> 0.05
CpG3	−0.032	0.063	> 0.05	−0.036	0.019	> 0.05	+0.005	0.037	> 0.05
CpG4	−0.414	0.166	< 0.05	−0.014	0.020	> 0.05	+0.015	0.036	> 0.05
CpG5	−0.073	0.060	> 0.05	−0.008	0.012	> 0.05	+0.003	0.035	> 0.05
CpG6	−0.219	0.076	< 0.05	−0.067	0.019	> 0.05	+0.018	0.036	> 0.05
CpG7	−0.021	0.081	> 0.05	−0.053	0.015	> 0.05	+0.003	0.039	> 0.05
CpG8							+0.016	0.039	> 0.05
Mean	−0.321	0.027	< 0.05	−0.031	0.016	> 0.05	+0.014	0.034	> 0.05

β, Standardized coefficients. SE, standard error.

## Discussion

Environmental contaminants are responsible for almost five million deaths and over eighty million disability-adjusted life years worldwide [23]. Heavy metal mercury derived from environmental pollution is listed in the top ten chemicals of public health concern by the WHO reports. Earlier animal experimental results from mouse [24], chick [25] and zebra fish [26] indicated that prenatal exposure to methylmercury induced NTDs. In addition, an epidemiological study reported that higher placental levels of Hg were associated with an elevated risk of NTDs [5], which supports a previous result that maternal elevated urinary Hg levels is associated with higher risk of NTDs [27]. These studies have indicated the impact of mercury on fetal development. However, most of published studies regarding the effects on growth and development have focused on their effects through maternal exposure. While potential effects through paternal germ line exposure are rarely examined. To explore the potential impact of paternal experience of exposure to Hg on offspring, in the present study, to our knowledge for the first time this study examined the correlation between urine internal Hg level and methylation level of the three imprinting genes *H19*, *Meg3* and *Peg3* in human sperm DNA. Interestingly, the results demonstrated a significant negative correlation between DNA methylation levels at *H19* DMR and Hg internal exposure level, implicating the ability of sperm responding to environmental conditions.

Recently, public exposures to low levels of mercury have received increased attention as a result of general environmental pollution. Many studies have shown that chronic exposure to Hg affected testosterone levels, disturbing the blood-testis barrier, and perturbing spermatogenesis from animal studies [8]. However, the molecular mechanisms of the toxicity remain to be understood, in particular lack of human data. In the present study, although the participants are non-occupational Hg exposure, the median concentration of urinary mercury in our samples were 9.14 μg/L, which is higher compared with previously reported urinary background levels of mercury (ranging from 0.25 to 9.0 μg/L) in the general population and unexposed workers in the different countries and regions in the world [28]. Moreover, 42.7% of urinary mercury in these participants is beyond normal level for non-occupational exposure population, which is not likely associated with whether aquatic products intake, implicating a chronic exposure of environmental Hg pollution. Importantly, although low levels of mercury exposure may not have detectable adverse alterations in the body, our results revealed a correlation between methylation level of imprinting gene *H19* and urinary Hg concentration, indicating its potential impact on gene expression. Birth defects are a leading cause of infant mortality in the world. Although the exact causes remain uncovered, the levels of exposure to toxins and environmental pollutants have been considered a direct effect on fetal development [29]. Recently, accumulating evidence has improved our understanding of the role of imprinting gene on regulating fetal growth and development. For example, 197 imprinted genes either experimentally validated or computationally predicted filtered from a publically available database were used for ingenuity pathway analysis, which enriched 57 imprinted genes in metals related biological function [30]. Previous studies have proposed that dynamic genomic imprinting modification as an adaptive model of developmental plasticity i.e., imprinted gene may be very susceptible to environmental perturbations and therefore play a key role in the plastic development regulates and control the function of the cellular through epigenetic modifications [31]. In the present study, our genes of interest include paternally imprinted genes *H19*, *Meg3* and maternally imprinted gene *Peg3*, and found that *H19* is most sensitive to Hg and resulted in an alteration of hypomethylation.

*H19* is known as one of the best characterized imprinted genes in human with varies functions. Particularly, methylation in DMRs of this imprinting gene plays a vital role on early embryonic development. The human H19 gene also encodes a 2.3 kb long, spliced, and polyadenylated non-coding RNA that plays important roles in embryonic development and growth [32]. Disturbance of methylation status in this gene, however, has been reported to lead to many congenital growth disorders. For instance, demethylation of *H19* was associated with growth-related syndromes in human: hypomethylation of *H19* being described in Silver-Russell Syndrome, characterized by severe intrauterine and postnatal growth retardation among other clinical features [33]. The study of samples from umbilical cord, reflecting maternal exposure to environmental adverse factors, indicated that reduced *H19*-DMR methylation was related to decreased head circumference in newborns [34]. Whereas in a case-control study, examination of stillborn brain tissue from diagnosed NTDs revealed the hypermethylation of *H19* compared with controls [35], which is not consistent with other studies. These results implicate that the role of *H19* is complicated along with different tissues, developmental stages and environmental factors. DMR of *H19* in both somatic and sperm DNAs in the male offspring has been reported to be very sensitive to environmental insults, such as it exhibited significant methylation percentage losses following alcohol administration in pregnant mice [22]. In our recent results from mice, methylation levels of sperm DNA of *H19* in F1-2 offspring show a similar pattern of hypomethylation as their benzo[a]pyrene (BaP) exposed F0 fathers (Zhang et al., to be published), supporting previous results of hypomethylation following exposure. Importantly, we present data of sperm in human with different urine Hg level in this study, that higher urine Hg was associated with decreased methylation level, in particular of the CpG 2, CpG4, CpG6 in the DMR of *H19*. The results provide indirect evidence that sperm DNA DMR of *H19* is sensitive to the environmental Hg exposure and suggest importance of methylation *H19* as a potential biomarker for fetal development. Dysregulation of imprinting at *Peg3* has been associated with growth disorders [36–38]. *Meg3* is a paternally imprinted gene. *Meg3* imprinting cluster is a critical region, established during maturation of sperm. In the present study, the average methylation level of *Meg3* instead of heavy methylated, as it suppose is, was in low level. Although the reason is not clear, a recent study found that there are non-imprinted regions of MEG3 in humans as well as a paternally imprinted secondary promoter in MEG3 [39]. Therefore, whether this is possible explanation for the reason is worth of further study. *MEG3* plays an essential role in expression regulation of Meg3 in the human body [40], where the locus-averaged methylation of the IG-DMR was reduced in fetal alcohol syndrome cases, which may potentially contribute to the growth and neuronal deficits in affected individuals [41]. However, in this study we fail to find any correlation between urine Hg concentration and the sperm DNA methylation in *Peg3* and *Meg3*. The reason may be due to different environmental insults targeting specific gene.

The studies of environmentally induced epigenetic modifications are currently expanding to paternal germ line that may provide an explanation for the transgenerational influence of father's experiences on offspring development. There is emerging literature focusing environmental influence on offspring development and fitness through paternal effects. For instance, a human epidemiological study indicated an increase in the rate of spontaneous abortions with an increasing concentration of fathers urinary Hg of prior to pregnancy [42], implicating a role of paternal Hg exposure on the offspring development. More recently, studies examined the association between preconception paternal alcohol exposure and somatic DNA of sired offspring. Significantly reduced DNA methylation at the CTCF binding sites at *H19* ICR of the offspring in mice [43] and an increase in DNA methylation at the *Peg3* DMR in sperm DNA through intragastric ethanol intubation was observed. Importantly, a similar changed pattern of methylation was found in the offspring [44]. All of these studies support that sperm DNA methylation patterns of imprinted genes are sensitive to environmental insults with dynamic characteristic, providing a means by which previous exposure experience can be “signatured” on chromosomes [45,46]. Although our results showed that the reduced sperm DNA methylation in *H19* correlates with an increase of urine Hg internal level, which may have adverse consequence on the embryo development [47] and on the offspring’s health, the timing and dose of these methylation alteration remain unknown. Due to many paternal effects on fetal development are mediated by maternal responses and mix with maternal effects, understanding the epigenetic plasticity in responding to environmental factor in the male germ cells contribute to understanding paternal effects on embryonic development as well as health outcomes of offspring. Further research is needed to evaluate the risk of epigenetic abnormalities induced by environmental insults for next generations in human and critical exposure.

## Supporting information

S1 Fig Schematic view of locations and sequences used by pyrosequencing in the DMR of the H19, Peg3 and Meg3 genes. TSS, transcription start site; DMR, differentially methylated region.
(TIF)

S1 Table KAP questionnaire of reproductive health in reproductive-aged men. The questionnaire presented here is a translation from S2 Table.
(DOC)

S2 Table KAP questionnaire of reproductive health in reproductive-aged men. This Chinese version questionnaire was actually used in this study.
(DOC)

S3 Table Social-demographic characteristics and urinary Hg levels in 242 subjects. An analysis was carried out with the 242 participants, who were included in the DNA methylation analysis. The results showed a similar relationship between Social-demographic characteristics and Urinary Hg Levels presented in Table 2 as the relationships for all subjects.
(DOC)

## Abbreviations

DMR s: differentially methylated regions
*MEG3*: maternally expressed gene 3
*PEG3*: paternally expressed gene 3
