# Supplementary figures and images for: Urine mercury levels correlate with DNA methylation of imprinting gene H19 in the sperm of reproductive-aged men
Source: PLoS One. 2018 Apr 26;13(4):e0196314. doi: 10.1371/journal.pone.0196314 (PMC5919660; doi:10.1371/journal.pone.0196314)

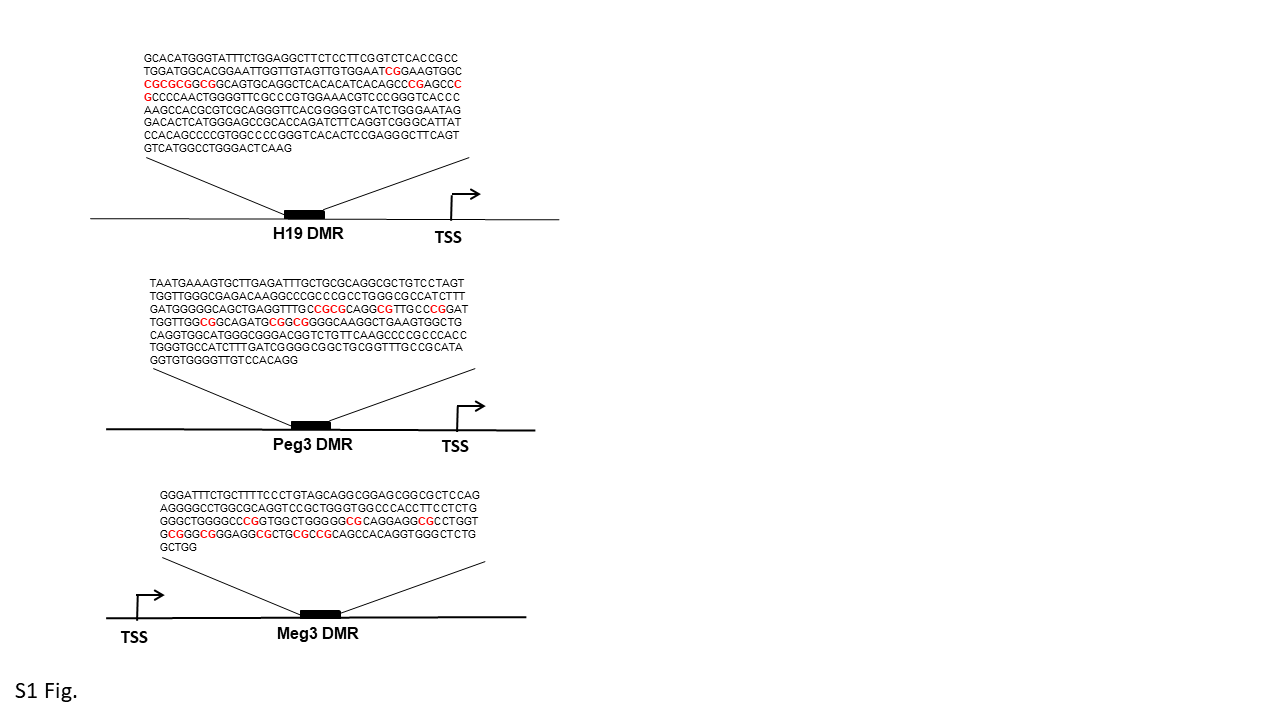

Supplement: S1 Fig — TSS, transcription start site; DMR, differentially methylated region. (TIF) [file pone.0196314.s001.tif]
