# Supplementary material for: Urine mercury levels correlate with DNA methylation of imprinting gene H19 in the sperm of reproductive-aged men
Source: PLoS One. 2018 Apr 26;13(4):e0196314. doi: 10.1371/journal.pone.0196314 (PMC5919660; doi:10.1371/journal.pone.0196314)
Supplement: S1 Table — The questionnaire presented here is a translation from S2 Table. (DOC) [file pone.0196314.s002.doc]

**Supplemental table 1 (S1 Table)**

**KAP questionnaire of reproductive health in reproductive-aged men**

**No:**

**1.** Demographic information**:**

Name： Address:

Purpose of visiting：

Telephone number：

A1 Date of Birth: Month Day_____ Year

A2 Education：①Primary school and below ②Middle school ③High school ④ College and higher

A3 Do you have family diseases history including birth defects?

1. No

② Yes（Explain: Relationship: ）

1. Lifestyle and dietary preference

D1 Do you smoke?

① No

② Yes (Smoking at least one cigarette per day on average for more than a year)

D3 Do you drink?

① No

② Yes (Drinking at least once per week on average for more than a year, including spirits, mild wine and beer. And Drinking 100 ml at a time.)

D17 Do you eat aquatic products?

1. No

② Yes (Eating aquatic products including any type of fish and seafood at least once per week on average for more than a year.)
