# Supplementary material for: Urine mercury levels correlate with DNA methylation of imprinting gene H19 in the sperm of reproductive-aged men
Source: PLoS One. 2018 Apr 26;13(4):e0196314. doi: 10.1371/journal.pone.0196314 (PMC5919660; doi:10.1371/journal.pone.0196314)
Supplement: S2 Table — This Chinese version questionnaire was actually used in this study. (DOC) [file pone.0196314.s003.doc]

**已婚育龄人群生育健康KAP调查 （男性）** 编号

**一. 人口学信息:**

姓名： 住址:

本次就诊的目的：

电话：

A1 出生日期: 年 月 日 (阳历)

A2 文化程度：①小学及以下 ②初中 ③高中（包括中专） ④大专及以上

A3 你家族是否有遗传病史：

无 有（详述 ）

A4 你家族是否有出生缺陷史：

无 有 （详述 与你关系 ）

A5 你家族是否有流产史：

无 有（与你的关系 ）

A6 你家族是否有不孕不育史：

无 有（与你的关系 ）

**二、生活方式**

D1你是否吸烟

①否

②是——平均每日吸烟１支或以上，且持续１年以上

D3你是否饮酒

①否

②是：平均每周至少饮酒１次（烈性酒、温和酒、啤酒），每次 2 两或以上，且持续１年以上

D17在日常饮食中你是否会注意水产品的摄入

①否 ②是：平均每周至少摄入水产品（包括各种海产品）１次，且持续1年以上
