# Supplementary material for: Urine mercury levels correlate with DNA methylation of imprinting gene H19 in the sperm of reproductive-aged men
Source: PLoS One. 2018 Apr 26;13(4):e0196314. doi: 10.1371/journal.pone.0196314 (PMC5919660; doi:10.1371/journal.pone.0196314)
Supplement: S3 Table — An analysis was carried out with the 242 participants, who were included in the DNA methylation analysis. The results showed a similar relationship between Social-demographic characteristics and Urinary Hg Levels presented in Table 2 as the relationships for all subjects. (DOC) [file pone.0196314.s004.doc]

**S3 Table. Social-demographic characteristics and Urinary Hg Levels in 242 subjects**

|  | N (%) | | Urinary Hg (μg/L)* | | | | | | p-values |
| --- | --- | --- | --- | --- | --- | --- | --- | --- | --- |
|  |  |  | Median | P_25_ ~ P_75_ | Min | Max | Over-criteria **  N (%) | |  |
| Age |  | |  |  |  |  |  | | > 0.05 |
| 22-24 | 2 (0.8) | | 8.30 | — | 6.55 | 10.05 | 1 (50) | |  |
| 25-29 | 78 (32.2) | | 9.66 | 4.93 ~ 13.66 | 0.16 | 39.15 | 37 (47.4) | |  |
| 30-35 | 115 (47.5) | | 8.86 | 4.17 ~ 13.46 | 0.73 | 71.35 | 49 (42.6) | |  |
| 36+ | 47 (19.4) | | 8.48 | 5.07 ~ 10.67 | 0.45 | 31.42 | 18 (38.3) | |  |
| Smoking | | | | | | | | |  |
| Yes | 141 (58.3) | | 9.34 | 4.96 ~ 13.50 | 0.45 | 71.35 | 66 (46.8) | | > 0.05 |
| No | 101 (41.7) | | 8.48 | 4.61 ~ 12.86 | 0.16 | 39.15 | 39 (38.6) | |  |
| Drinking | | | | | | | | |  |
| Yes | 79 (32.6) | | 8.76 | 4.87 ~ 13.57 | 0.45 | 71.35 | 33 (41.8) | | > 0.05 |
| No | 163 (67.4) | | 9.03 | 4.64 ~ 13.17 | 0.16 | 48.47 | 72 (44.2) | |  |
| Aquatic products intake |  |  |  |  |  |  |  |  |  |
| Yes | 120 (49.6) | | 9.28 | 4.43 ~ 13.28 | 0.16 | 59.22 | 56 (46.7) | | > 0.05 |
| No | 122 (50.4) | | 8.46 | 5.07 ~ 12.98 | 0.45 | 71.35 | 49 (40.2) | |  |
| Education | | | | | | | | |  |
| Primary school | 2 (0.8) | | 19.93 | — | 8.44 | 31.42 | 1 (50) | | > 0.05 |
| Middle school | 61 (25.2) | | 9.48 | 4.57 ~ 13.30 | 0.38 | 30.41 | 28 (45.9) | |  |
| High school graduate | 52 (21.5) | | 9.24 | 5.31 ~ 15.07 | 0.45 | 71.35 | 25 (48.1) | |  |
| College or above | 127 (52.5) | | 8.48 | 4.35 ~ 12.94 | 0.16 | 48.47 | 51 (40.2) | |  |
| Total | 242 (100) | | 8.90 | 4.83 ~ 13.17 | 0.16 | 71.35 | 105 (43.4) | |  |

*Normalized by common specific gravity.

**Criterion of Hg intoxication: urinary Hg concentration ≥10 μg/L. US CDC. Specific Hazards/Chemical mergencies/ Mercury. Available from URL: https://emergency.cdc.gov/agent/mercury/mercelementalcasedef.asp
